# Integument cell differentiation in dandelions (*Taraxacum*, Asteraceae, Lactuceae) with special attention paid to plasmodesmata

**DOI:** 10.1007/s00709-015-0894-2

**Published:** 2015-10-10

**Authors:** Bartosz J. Płachno, Ewa Kurczyńska, Piotr Świątek

**Affiliations:** 1 Department of Plant Cytology and Embryology, Jagiellonian University in Kraków, 9 Gronostajowa St., 30-387 Kraków, Poland; 2 Department of Cell Biology, University of Silesia, 28 Jagiellońska St., 40-032 Katowice, Poland; 3 Department of Animal Histology and Embryology, University of Silesia, 9 Bankowa St., 40-007 Katowice, Poland

**Keywords:** Apomixis, Asteraceae, Cell wall, Integument, Mucilage, Ovule, Plasmodesmata, Ultrastructure

## Abstract

The aim of the paper is to determine what happens with plasmodesmata when mucilage is secreted into the periplasmic space in plant cells. Ultrastructural analysis of the periendothelial zone mucilage cells was performed on examples of the ovule tissues of several sexual and apomictic *Taraxacum* species. The cytoplasm of the periendothelial zone cells was dense, filled by numerous organelles and profiles of rough endoplasmic reticulum and active Golgi dictyosomes with vesicles that contained fibrillar material. At the beginning of the differentiation process of the periendothelial zone, the cells were connected by primary plasmodesmata. However, during the differentiation and the thickening of the cell walls (mucilage deposition), the plasmodesmata become elongated and associated with cytoplasmic bridges. The cytoplasmic bridges may connect the protoplast to the plasmodesmata through the mucilage layers in order to maintain cell-to-cell communication during the differentiation of the periendothelial zone cells.

## Introduction

In some members of the Asteraceae family, there is a specific cell differentiation in the integument. The part of the internal integument behind the endothelium and the region near the antipodal cells form a zone that is called the periendothelial zone and that is also called the nutritive integument tissue or pseudochalaza (Cooper and Brink 1949; Engell and Petersen 1977; Pandey et al. 1978; Musiał et al. 2013). Depending on the changes in this integument zone, three types of ovules were proposed in Asteraceae: “Taraxacum”, “Galinsoga” and “Ratibida”. “Taraxacum” type cells have very swollen walls with a spongy structure (Kolczyk et al. 2014). Some characteristics of these cells such as a reduction in protoplast size and thickening of the cell wall are similar to mucilage cells. According to Pandey et al. (1978), the cells of periendothelial zone undergo gelatinisation in *Youngia japonica*. Moreover, a liquefaction of the integument cells was observed in *Hieracium* by Koltunow et al. (1998). Musiał et al. (2013) claimed that these cells undergo progressive degeneration in *Taraxacum*, which is probably by programmed cell death (PCD).

In plants, mucilage may play various physiological roles: carbohydrate storage (Pimienta-Barrios and Nobel 1998), catching prey in carnivorous plants with adhesive traps (e.g., Juniper et al. 1989; Król et al. 2012), water storage and drought tolerance (Clifford et al. 2002), frost tolerance (Lipp et al. 1994) and host-pathogen interactions (Davis et al. 1986). The role of mucilage cells in ensuring seed viability and germination (Western et al. 2000; Yang et al. 2010; Kreitschitz 2012) may apply to *Taraxacum* ovules and young seeds.

In recent years, it has been postulated that the symplasmic communication that occurs due to the existence of plasmodesmata in plants plays an important role in the regulation of cell differentiation (Brunkard et al. 2013; Burch-Smith et al. 2011; Marzec and Kurczynska 2014), since it was shown that plasmodesmata provide a passageway not only for nutrients but also for various types of signal molecules including proteins, transcriptional factors and RNAs (Gursanscky et al. 2011; Hyun et al. 2011; Jang 2014). The correlation between symplasmic communication and cell differentiation was shown for different developmental processes such as zygotic and somatic embryogenesis (Kozieradzka-Kiszkurno et al. 2011; Kozieradzka-Kiszkurno and Płachno 2012), androgenesis (Wrobel et al. 2011), root cell differentiation (Benitez-Alfonso et al. 2014; Marzec et al. 2013), shoot apical meristem organisation and transition from a vegetative to generative state of development (Paul et al. 2014) or the development of cambial cells (Ehlers and van Bel 2010; Fuchs et al. 2010). Plasmodesmata are dynamic “intercellular bridges” whose permeability to different molecules changes according to the state of development or the influence of different signals, both external and internal (Kragler 2013; Marzec and Kurczynska 2014; Ueki and Citovsky 2011). Changes in plasmodesmata permeability are correlated with their modifications such as elongation, branching, fusion or even loss of appressed ER (Kollmann R. Glockamnn C 1999). Such modifications were described, for example, in the case of the Strasburger cell in *Abutilon* nectar trichomes or in the parenchyma sheet of maize leaves and during the development of wheat caryopses (Glockmann and Kollmann 1996). In the mucilage cells of dicotyledons, the secreted material is deposited between the plasmalemma and the cell wall (Bakker and Baas 1993). The mucilage accumulates in the periplasmic space, thus pressing the protoplast away from the cell wall, and, according to Baker and Baas (1993), the mucilage covers the plasmodesmata, which leads to the loss of plasmodesmata function and cell-to-cell communication.

However, our knowledge about plasmodesmata modification and symplasmic transport in such a cell type is still insufficient. The question is what happens when material is secreted into the periplasmic space in *Taraxacum*: (1) Do plasmodesmata become sealed (occluded by wall material) or degraded or (2) Do they undergo other modifications?

To the best of our knowledge, we describe the differentiation of plasmodesmatal connections in the mucilage cells of angiosperms in detail for the first time. The paper presents the unusual structure of the cell wall and the differentiation of plasmodesmatal connections in the periendothelial zone of *Taraxacum. Taraxacum* is the model taxon for apomixis and its integument tissue was shown to influence apomictic development (Tucker et al. 2012). Thus, our observations on the differentiation of the integument tissue in this taxon may contribute to our understanding of sexual and apomictic seed development under the influence of the integument.

## Material and methods

### Plant material

We studied and compared the changes in ovule tissue among several sexual and apomictic *Taraxacum* species. Sexual species were represented by two diploid species—*Taraxacum tenuifolium* (Hoppe & Hornsch.) Koch (*T*. sect. *Palustria*) and *Taraxacum linearisquameum* Soest [*T*. sect. *Taraxacum*; syn *T*. sect. *Ruderalia* (Kirschner and Štěpánek 2011)]. The apomictic sample was represented by various apomictic taxa from the *T*. sect. *Taraxacum* (*T. officinale* agg. clone SA-B from Palacký University, Olomouc, Czech Republic and specimens that were collected in Kraków-Podgórze, Poland) and *Taraxacum brandenburgicum* Hudziok (sect. *Palustria*). We also used *Taraxacum pieninicum* Pawł. (section *Erythrocarpa*) from the Botanical Garden of the Maria Curie-Skłodowska University, Lublin collection. Studies were carried out on buds and flowers before and during anthesis.

Irrespective of the time of harvest before or during anthesis, the flowers of the diploid species contained ovules with mature embryo sacs of the Polygonum type. The flowers of the apomictic species harvested before anthesis contained the mature embryo sac whereas the flowers harvested during anthesis already contained embryo and endosperm, as previously observed (Płachno et al. 2014; Płachno et al. 2015). Analysis was performed on at least three different samples from each species. Sixteen sections were analysed in TEM (about ten mucilage cells per section were analysed in TEM for each ovule and ten other typical integumentary cells were also analysed for comparison). About 160 walls with plasmodesmata of mucilage cells were analysed. The figures that are presented are representative of all of the species that were analysed.

Vouchers of the taxa that were examined were deposited in the herbarium of Jagiellonian University in Kraków (KRA), with the exception of *T. officinale s.l*. (clone SA-B), which was deposited in the herbarium of the Department of Botany, Palacký University, Olomouc, Czech Republic.

### Light and electron microscopy studies

The preparation of the samples for TEM (transmission electron microscopy) followed the procedure described by Płachno and Świątek (2009) and Płachno (2011). Briefly, ovaries were fixed in 2.5 % formaldehyde and 2.5 % glutaraldehyde in a 0.05 M cacodylate buffer (pH 7.0) for 2 days. The material was postfixed in 1 % OsO_4_ in a cacodylate buffer for 24 h at ∼4 °C, rinsed in the same buffer, treated with 1 % uranyl acetate in distilled water for 1 h, dehydrated with an acetone series and embedded in an Epoxy Embedding Medium Kit (Fluka) or in Spurr’s resin. Semithin sections were stained using aqueous methylene blue with azure II for general histology (Humphrey and Pittman 1974) for 1–2 min (MB/AII) and examined using an Olympus BX60 microscope. The periodic acid-Schiff (PAS) reaction was used to visualise the total carbohydrates of insoluble polysaccharides (Wędzony 1996). The presence of mucilage substances was analysed using neutral red according to the method described by O’Brien and McCully (1981).

Ultrathin sections were cut on a Leica Ultracut UCT ultramicrotome. After contrasting with uranyl acetate and lead citrate, the sections were examined using a Hitachi H500 electron microscope at 75 kV in the Faculty of Biology and Environmental Protection, University of Silesia in Katowice and a Jeol JEM 100 SX; JEOL, Tokyo, Japan, at 80 kV in the Department of Cell Biology and Imaging, Institute of Zoology, Jagiellonian University in Kraków.

## Results

**Fig 1:**
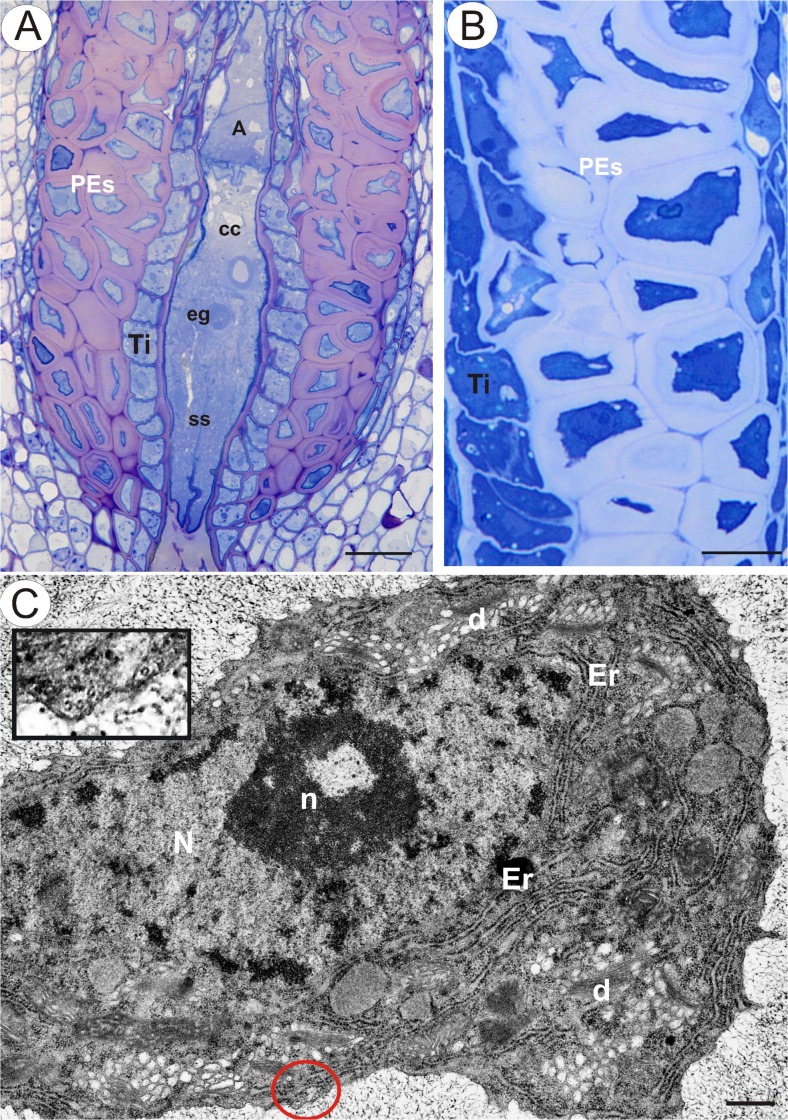
**a** *T. officinale s.l*. (clone SA-B). Semithin section through an ovule with embryo sac showing the structure of periendothelial zone cells (*PEs*); egg cell (*eg*), central cell (*cc*), synergids (*ss*), antipodes (*A*) and integumental tapetum (*Ti*), *bar* = 20 μm. **b** *T. officinale s.l*. (clone SA-B). Semithin section through the integument showing periendothelial zone cells, *bar* = 10 μm. **c** Sexual dandelion *T. tenuifolium* (ovule from flower during anthesis). Ultrastructure of periendothelial zone cells; dictyosomes (*d*), rough endoplasmic reticulum (*Er*), nucleus (*N*), nucleolus (*n*) and framed part shows cortical microtubules near plasmalemma (*red circle*), *bar* = 0.5 μm

**Fig 2:**
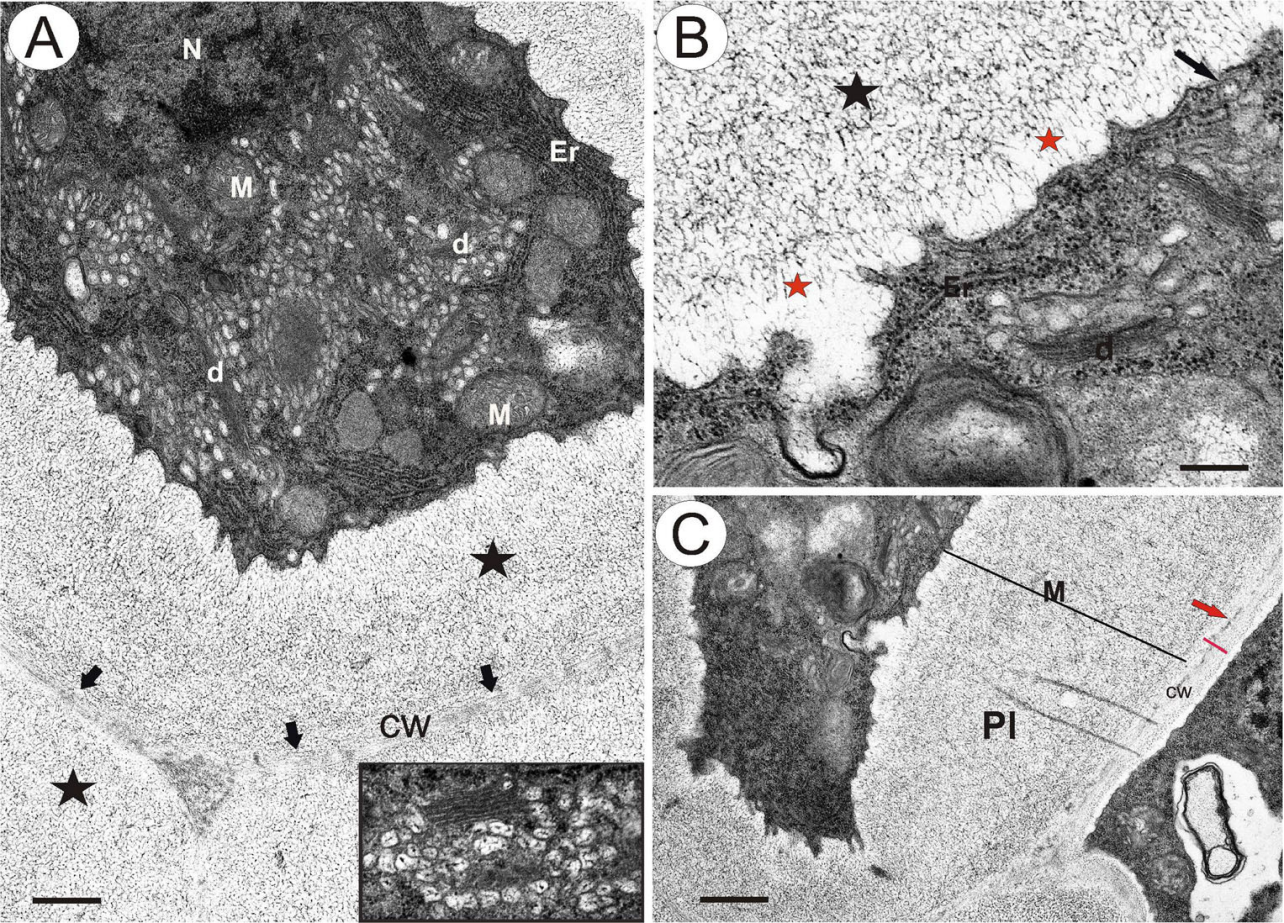
Ultrastructure of periendothelial zone cells in *Taraxacum*. **a** *T. officinale s.l*. (clone SA-B, ovules from flowers just before anthesis) electron micrograph showing the general ultrastructure of a periendothelial zone cell; mitochondrion (*M*), dictyosomes (*d*), rough endoplasmic reticulum (*Er*), nucleus (*N*), primary cell wall (*cw*, *arrows*) and cell wall layer with a reticulate appearance of wall material (*star*), *bar* = 0.6 μm; framed part shows *T. tenuifolium* dictyosome with numerous vesicles that contain fibril material. **b** The *T. officinale s.l*. (clone SA-B) part of a periendothelial zone cell, note the lobed plasmalemma, cortical microtubules and exocytose of the dictyosome vesicle (*arrow*), dictyosome (*d*), rough endoplasmic reticulum (*Er*), amorphic layer of the cell wall (*red star*) and the cell wall layer with microfibrils (*star*), *bar* = 0.3 μm. **c** *T. officinale s.l*. (clone SA-B) plasmodesmata (*Pl*) between a periendothelial zone cell and parenchyma cell, reticulate mucilage material (*M*), middle lamella (*arrow*) and primary cell wall (*cw*, *red line*), *bar* = 1 μm

In samples showing mature embryo sacs (in apomicts in closed flowers; see Fig. 1a, in sexual dandelions during anthesis), the cells of periendothelial zone are still alive, despite the reduction in protoplast size (Fig. 1b, c). At this stage, the cytoplasm of the periendothelial zone cells is dense and is filled by numerous organelles. The protoplast has an irregular shape with numerous plasmalemma invaginations. Cortical microtubules are clearly visible near the plasmalemma (Fig. 1c). There are numerous profiles of rough endoplasmic reticulum and dictyosomes with numerous vesicles that are present mostly in association with the newly formed cell walls (Figs. 1c and 2a). The dictyosome vesicles are large and contain fibrillar material (Fig. 2a). Exocytosis of these vesicles was observed (Fig. 2b). The nuclei and nucleoli of the periendothelial zone cells are large (Fig. 1c). Staining with methylene blue with azure II allows different layers in the wall of periendothelial zone cells to be distinguished (Fig. 1b). In addition, the layers are clearly visible in TEM (Fig. 2a). In the vicinity of the plasmalemma, there was an almost amorphous, electron-translucent layer that had fibrillar, electron-dense components (Fig. 2b). Moreover, a thick layer of an amorphous wall matrix with dense fibrillar material in a reticulate arrangement (Fig. 2a, b) and the primary cell wall, which was present before the deposition of the mucilage material, were distinguished (arrows, Fig. 2b). The thickening of the cell wall apparently occurs through the apposition of secreted material. Staining with neutral red and with methylene blue with azure II showed that the first two layers had a mucilage character. A middle lamella was seen as an electron-dense layer, especially between the periendothelial zone cells and other integument cell types (Fig. 2c).

**Fig 3:**
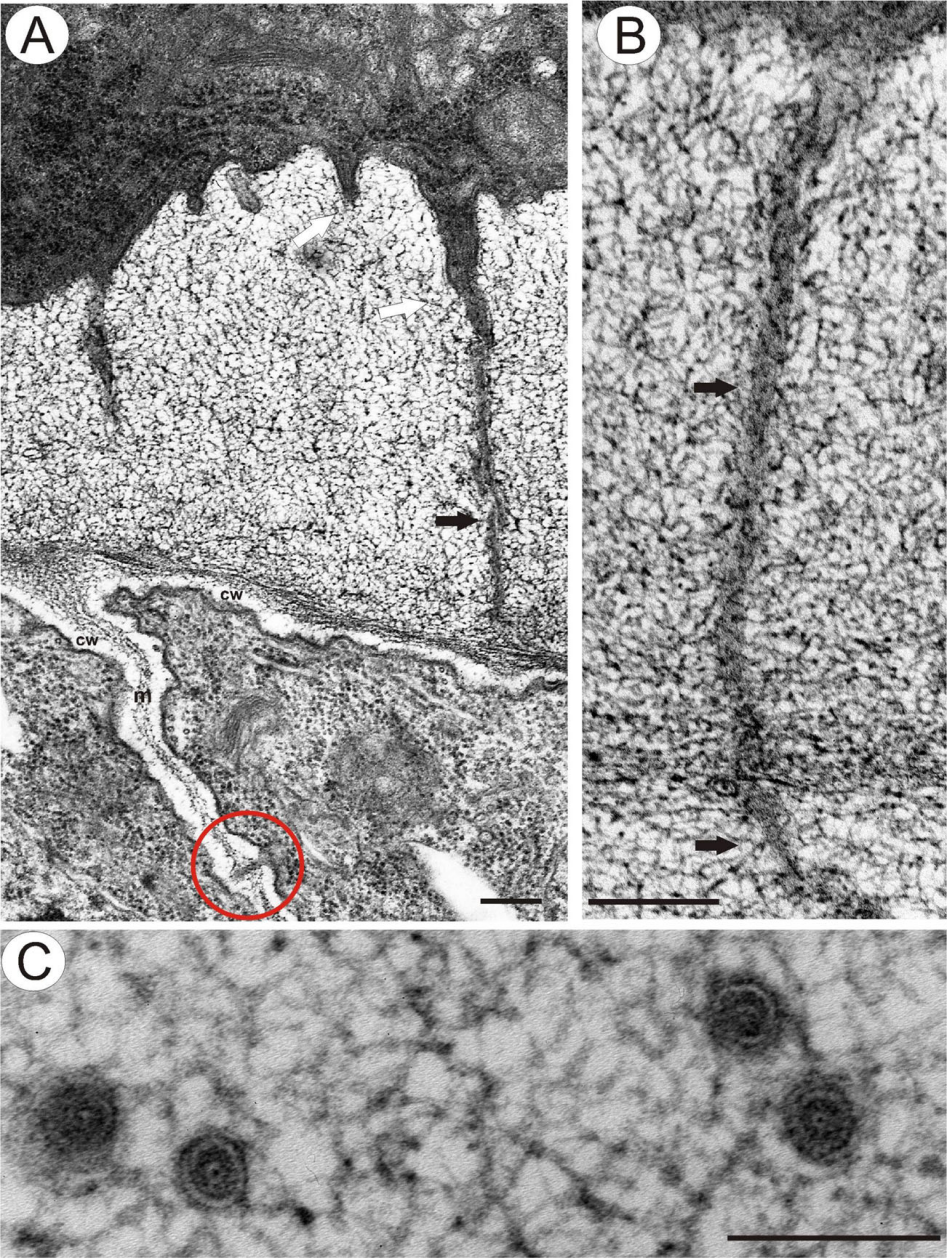
Plasmodesmata and cytoplasmic bridges in the integument cells of *T. officinale s.l*. (clone SA-B, ovules from flowers just before anthesis) **a** Contact between a periendothelial zone cell and parenchyma cell, cytoplasmic bridges (*white arrow*), cytoplasmic connections (*black arrow*) in reticulate appearance mucilage material, plasmodesma in the primary cell wall (in *red circle*), middle lamella (*m*) and primary wall (*cw*), *bar* = 0.25 μm. **b** Longitudinal section though cytoplasmic connections (*arrow*) shown in **a**, *bar* = 0.2 μm. **c** Transverse section through plasmodesmata in this modified wall close to the primary wall, *bar* = 0.1 μm

**Fig 4:**
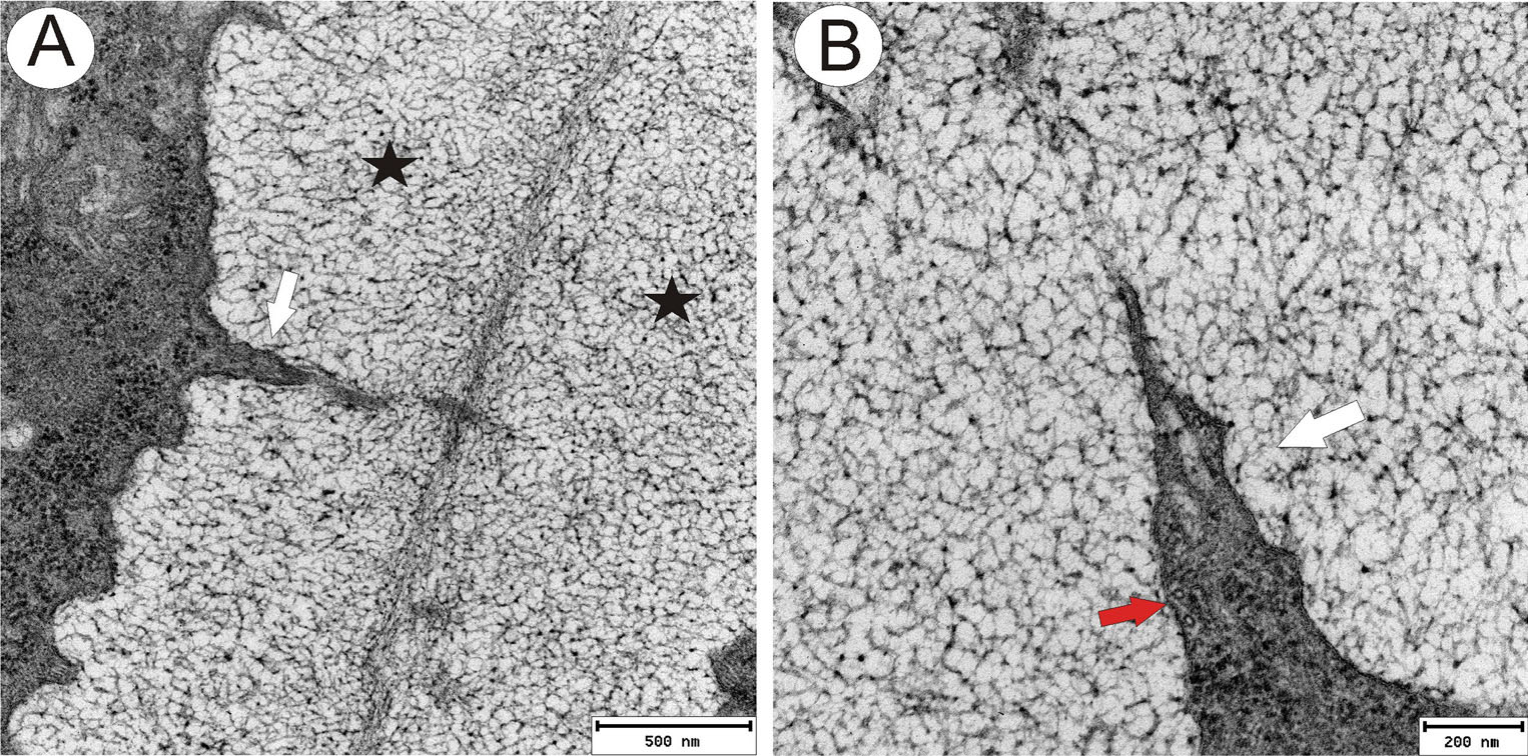
Cytoplasmic connections in the integument cells of *T. officinale s.l*. (clone SA-B, ovules from flowers just before anthesis) **a**, **b** Longitudinal section though cytoplasmic connections in the mucilage material (*white arrow*), modified wall - mucilage (*black star*), microtubules (*red arrow*), bar = 0.5 μm and bar = 0.2 μm

**Fig. 5:**
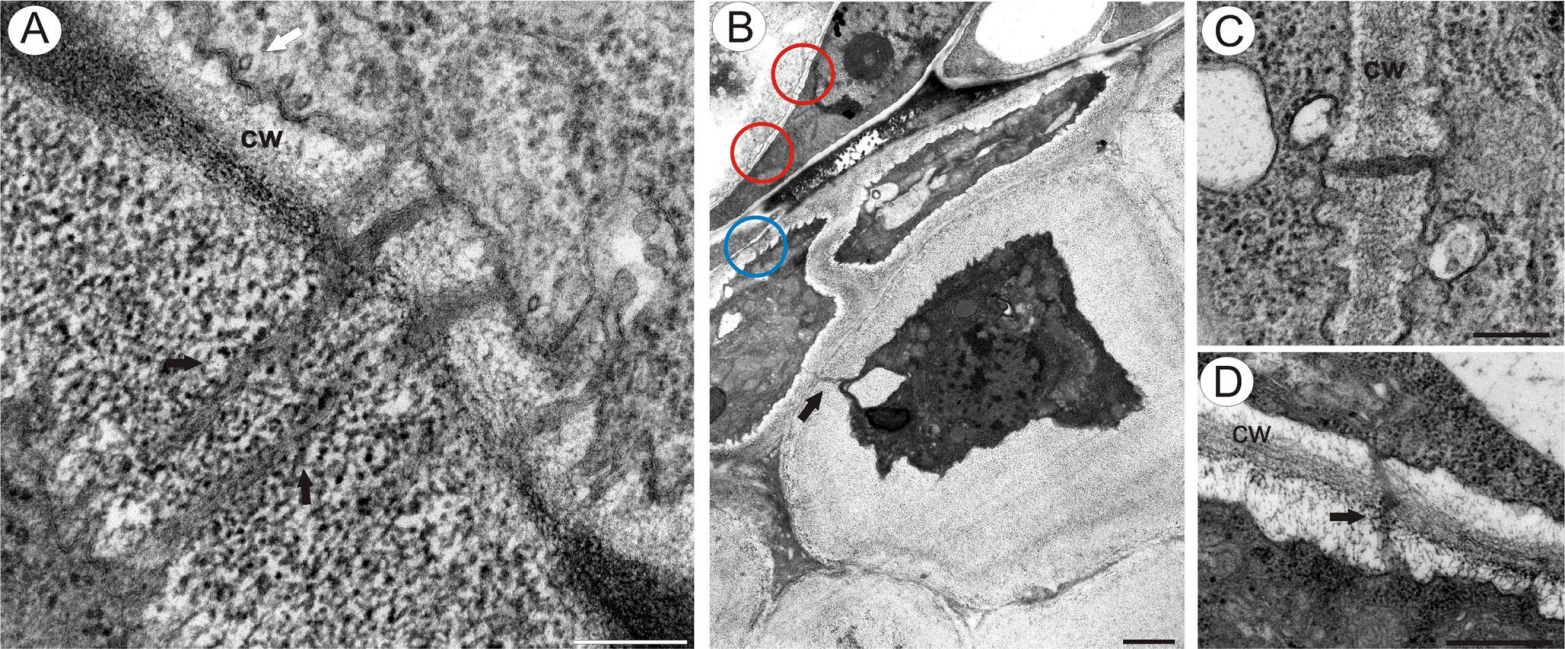
Plasmodesmata in integument cells. **a**–**d** *T. officinale s.l*. (clone SA-B, ovules from flowers just before anthesis); **a** Longitudinal section though plasmodesmata in the mucilage material (*black arrow*), primary wall (*cw*), microtubules (*white arrow*), *bar* = 0.2 μm. **b** Gradient of plasmodesmata modifications and mucilage deposition between different integument cells, typical plasmodesmata (*red circle*), slightly elongated plasmodesmata (*blue circle*), elongated plasmodesmata with a cytoplasmic connection in the mucilage layer (*arrow*), *bar* = 1.7 μm. **c** Typical plasmodesmata (similar ones are also shown in the *red circle* in (**b**)), primary wall (*cw*), *bar* = 0.2 μm. **d** Slightly elongated plasmodesma (*black arrow*) (similar ones are also shown in the *blue circle* in (**b**)), primary wall (*cw*), *bar* = 0.5 μm

Analysis of the modified wall ultrastructure showed the presence of electron-dense structures that traverse the mucilage layers and that may connect the cytoplasm to the plasmodesmata (Figs. 2c, 3a, b, 4a, b and 5a). These structures appeared to be cytoplasmic bridges (Fig. 4a, b) with clearly visible plasmalemma, microtubules and vesicles. Both their dimensions and the presence of microtubules inside these structures clearly indicate that these bridges are indeed cytoplasmic protrusions and do not represent extensions of the plasmodesmata themselves. However, typical plasmodesmata were present close to the primary wall in this modified wall (Fig. 3c). In some cases, these plasmodesmata traverse the wall perpendicular to the plane of the section (Fig. 3c). Their structure is typical with clearly visible plasmalemma and a desmotubule (Fig. 3c). There is a gradient of mucilage deposition that is dependent on the cell position (Figs. 3a and 5b–d). Periendothelial zone cells start to differentiate at the young ovule stage (when the young embryo sac occurs), and these cells have typical plasmodesmata in their cell walls. In fully developed mucilage periendothelial cells, mucilage material was deposited between the primary cell wall and plasmalemma on all sides of the cells (Fig. 2a). The cytoplasmic bridges only occurred in these kinds of cells. It appears likely that the cytoplasmic bridges allow the cells to maintain intercellular connectivity despite the formation of the large mucilage layers that spatially separate the protoplasts from the cell walls. One hundred percent of the plasmodesmata that were observed in these cells were connected with cytoplasmic bridges. Non-mucilage integumentary cells had typical plasmodesmata without cytoplasmic bridges (Fig. 5b, c). When a mucilage cell borders with a non-mucilage cell, the elongation of the plasmodesmata and the formation of cytoplasmic bridges occurred only from the side of the mucilage cell (Figs. 3a and 5a).

## Discussion

Only a few papers have been published that discussed results concerning the ultrastructure of the integumentary cells of Asteraceae (Newcomb 1973; Figueiredo et al. 2006; Musiał et al. 2013; Kolczyk et al. 2014). In the present paper, we show that the periendothelial cells in *Taraxacum* are rich in profiles of rough endoplasmic reticulum and dictyosomes with numerous secretory vesicles. Our ultrastructural observations in *Taraxacum* are similar to the results that were obtained by Newcomb (1973) in *Helianthus annus*. Newcomb observed that sunflower periendothelial zone cells were completely filled with wall material. This wall in *Taraxacum* was previously described as a wall with an open spongy structure by Musiał et al. (2013). A similar observation was made in other Asteraceae that have a *Taraxacum* type of ovule such as *Solidago*, *Chondilla*, and *Bellis* (Kolczyk et al. 2014). However, based on our observations, we now know that this new “wall material” has a mucilage character. This was suggested by both the texture of the deposited wall material as well as the staining reaction of this material in the presence of methylene blue with azure II and neutral red. This result corresponds well with the high activity of Golgi apparatus (numerous secretory vesicles with mucilage material), which was observed in our study on *Taraxacum* and also, previously, in *Helianthus* (Newcomb 1973). Moreover, we observed fibrillar material, which structurally resembles the fibrillar material deposited within the wall, in the Golgi vesicles. The Golgi apparatus was described as the source of mucilage in many types of mucilage cells in members of various families, e.g., Cactaceae, Droseraceae, Lauraceae and Malvaceae (Mollenhauer 1967; Outenreath and Dauwalder 1982; Trachtenberg and Fahn 1981; Bakker and Baas 1993). However, in some rare cases, other organelles are probably the source of mucilage as was proposed for *Aloe* (Trachtenberg 1984).

The integumentary mucilage cells of *Taraxacum* are similar in some characters to the mucilage cells of other dicotyledons in which mucilage material is deposited between the plasmalemma and the cell wall (Bakker and Baas 1993). However, the mucilage cells in these species are quite different than those that have been described in *Araucaria angustifolia* mesophyll (Mastroberti and de Araujo Mariath 2008). In the case of *Araucaria*, the secretion is deposited into a specialised cavity that is situated between the cytoplasm and the central vacuole (see Fig. 27 in Mastroberti and de Araujo Mariath 2008). However, like in *Taraxacum*, the mucilage structure in *Cinnamomum* (Bakker et al. 1991) or the gymnosperms *Araucaria* (Mastroberti and de Araujo Mariath 2008) showed a similar fibrillar or reticulate character. Thus, a Taraxacum-like mucilage occurs also in non-related taxa.

We observed that the periendothelial zone cells in *Taraxacum* begin to differentiate at the young ovule stage (when the young non-cellular embryo sac occurs). In *Helianthus*, this material deposition process begins during the early cellular embryo sac stage (Newcomb 1973). In *Hieracium*, the wall changes of the periendothelial zone cells start during the four-nucleate embryo sac stage (Koltunow et al. 1998).

Plasmodesmata undergo different modifications during cell differentiation (Jones 1976; Lucas and Wolf 1993). In the case of primary plasmodesmata, these modifications include elongation, branching or fusion of adjacent plasmodesmata (Kollmann R. Glockamnn C 1999). According to the definition, primary plasmodesmata are formed during cytokinesis. Primary plasmodesmata that undergo branching during the secondary apposition of wall material are called secondarily modified primary plasmodesmata. In contrast, secondary plasmodesmata are plasmodesmata that are formed across pre-existing cell walls (Botha and Cross 2001). In the presented studies, it is shown that the analysed mucilage cells are connected by primary plasmodesmata with a single linear channel. As the wall grows in thickness, these plasmodesmata grow in length, without undergoing other detectable modifications (Fig. 3c). Occlusion or closing of plasmodesmata in mucilage cells was suggested by early studies (Bakker and Baas 1993). However, our observation that the plasmodesmata in mucilage cells are linked to the protoplast via cytoplasmic bridges suggests that they are functional. An elongation of the primary plasmodesmata that correlated with an increase of the wall thickness was described for the Strasburger cells of the needles of *Metasequoia* (Glockmann and Kollmann 1996). The cytoplasmic strands may be analogous to Hechtian strands. Both Hechtian strands and cytoplasmic bridges connect plasmodesmata with the cytoplasm—in the case of Hechtian strands across the space caused by plasmolysis (Oparka et al. 1994) and in the case of the structures described in this paper across a region of mucilage.

### The function and fate of integumentary mucilage cells

It is believed that the periendothelial zone cells in Asteraceae nourish the embryo, thereby reducing the importance of the endosperm function (Koltunow et al. 1998; van Baarlen et al. 1999; Musiał et al. 2013). However, we think that in addition to a nutritional role (which should be experimentally proven), the mucilage periendothelial zone cells may facilitate the regulation of hydration during the early stages of seed development. Moreover, the degradation of these cells forms the space that is needed for the growing embryo. Degradation of this tissue during embryogenesis in *Taraxacum* was recorded by Cooper and Brink (1949). Figueiredo et al. (2006) observed PCD in the periendothelial zone cells of *Cynara*; however, they interpreted these cells as having a nucellar origin.

When we interpret the results about ovules in Asteraceae (Kolczyk et al. 2014) in the light of our results, it seems that the mucilage cells are a common character in Asteraceae ovules and occur in species that have a high reproductive success (genera *Taraxacum*, *Solidago*, *Chondilla*, *Bellis*). Thus, the mucilage cells seem to be important for the physiology of ovules and young seeds. The functional significance of the observed cytoplasmic bridges remains to be shown. We suppose that for active mucilage production, symplasmic transport is required. In order to ensure the proper function of plasmodesmata in these specialised cells, cytoplasmic bridges are formed.

## Conclusion

We show that specialised mucilage cells in the integument of developing *Taraxacum* seeds form cytoplasmic bridges to connect the cytoplasm with the plasmodesmata through mucilage layers. This suggests the importance of communication between these cells during seed development. However, further studies are needed to demonstrate that the cytoplasmic bridges are indeed required for intercellular communication in integument tissues.

### Future prospective

The plasmodesmata and cell contacts that are described provide an attractive model to test cell-to-cell communication during cell differentiation. Future analyses should involve studies on symplasmic transport and fluorochrome distribution within and between the integumentary tissues. Moreover, the chemical composition of the mucilage and the wall of the integument cells in *Taraxacum* must be studied using immunolabelling methods.
